# Two Novel Loci of *RELN* Associated With Antipsychotics Response in Chinese Han Population

**DOI:** 10.3389/fphar.2020.00007

**Published:** 2020-01-31

**Authors:** Qingqing Xu, Mo Li, Shengying Qin, Yaojing Li, Ailing Ning, Yingmei Fu, Dongxiang Wang, Duan Zeng, Huafang Li, Wenjuan Yu, Shunying Yu

**Affiliations:** ^1^ Shanghai Key Laboratory of Psychotic Disorders, Shanghai Mental Health Center, Shanghai Jiao Tong University School of Medicine, Shanghai, China; ^2^ Bio-X Institutes, Key Laboratory for the Genetics of Developmental and Neuropsychiatric Disorders, Ministry of Education, Shanghai Jiao Tong University, Shanghai, China; ^3^ Clinical Research Center, Shanghai Jiao Tong University School of Medicine, Shanghai, China; ^4^ Shanghai Mental Health Center, Shanghai Jiao Tong University School of Medicine, Shanghai, China

**Keywords:** schizophrenia, RELN, antipsychotics, drug response, pharmacogenetics

## Abstract

**Background:**

There are great individual differences in the drug responses; however, there are few prognostic drug response biomarkers available. *RELN* is one of the more extensively examined schizophrenia candidate genes. The purpose of this study was to determine whether *RELN* can affect antipsychotics response in the Chinese population. This may lead to the discovery of relevant novel drug response markers.

**Methods:**

The unrelated 260 Chinese Han inpatients with schizophrenia were enrolled in the present study. The enrolled subjects have been prescribed antipsychotic medication during the study. A total of 15 SNPs of *RELN* were genotyped by MassARRAY^®^ platform. The association of the *RELN* gene with therapeutic response to antipsychotics was analyzed based on sex and age at onset.

**Results:**

Two novel SNPs of *RELN* were found to be associated with antipsychotic treatment response (rs155333, p = 0.010 and rs6465938, p = 0.049) at nominal significance threshold, but not after multiple correction. Our study also revealed highly significant association of a haplotype consisting of three SNPs (rs362814-rs362626-rs2237628) with antipsychotic treatment response. Even after permutation, the *p*-value indicated significant association (rs362814-rs362626-rs2237628: ACT, χ^2^ = 6.353, *p* = 0.0117, permuted *p* = 0.04). Furthermore, a novel SNP, rs2535764, was found to be associated with antipsychotic response under overdominant genetic model at a marginal significant level of 0.046 (C/T vs. C/C + T/T: *p* = 0.046, AIC = 314.7, BIC = 321.6).

**Conclusion:**

Our data indicated that *RELN* can affect antipsychotic treatment outcomes in the Chinese population. SNPs of *RELN* could be used as predictive biomarkers for future personalized medicine of antipsychotic drug treatment. However, none of the three novel SNPs (rs155333, rs6465938, and rs2535764) remained significant after Bonferroni correction. Therefore, validation is needed in larger pharmacogenetic studies.

## Introduction 

Schizophrenia is a widespread mental disorder with periods of remission and relapses over a patient’s lifetime (van Os and Kapur, 2009; Jann and Penzak, 2018), and long-term treatment with antipsychotic drugs is often required (Kahn et al., 2015). The lifetime prevalence of schizophrenia in Chinese population is 0.9% (Huang et al., 2019). There are great individual differences in antipsychotic drug treatment responses, in terms of both therapeutic effects and adverse effects (Xu et al., 2013). For example, recent studies have shown that response rates for currently available antipsychotic drug treatment of first-episode psychosis are usually only about 50%–60% (Arranz and de Leon, 2007; Kahn et al., 2008; Robinson et al., 2015). Treatment failure of schizophrenia not only could increase economic costs but also could cause severe adverse drug reactions in patients.

In clinical practice, there is a lack of reliable predictors for antipsychotic drug responses, and currently no biomarker is available to guide medication. Risperidone, quetiapine, aripiprazole, olanzapine, and perphenazine are the most commonly used antipsychotic drugs in China. As with all antipsychotics, there are considerable individual differences in response to these drugs, in terms of both therapeutic effects and adverse effects. It has been reported that genetic polymorphism plays an important role in individual differences. Pharmacogenomics aims to identify biomarkers to maximize medication efficacy and minimize potential adverse events (Kirchheiner et al., 2005). In recent years, many studies have investigated the association between genetic variation and different antipsychotic drug responses (Xing et al., 2006; Fijal et al., 2009; Lee et al., 2012). However, the pharmacogenetics study of schizophrenia is still in its infancy. Most of the previous studies focused on genes related to the mechanisms of drug action. Many of these studies mainly focused on genes encoding drug targets (*e.g*., dopamine or serotonin receptors), drug transporters, and cytochrome P450 genes. This may omit other potentially significant antipsychotic drug response markers which are beyond these candidate genes. More exploratory studies that investigate other genes of interest likely represent a superior strategy for discovering relevant novel antipsychotic drug response markers. Moreover, these newly discovered antipsychotic treatment markers have the potential to be novel drug targets for schizophrenia.

*RELN* is an extensively studied schizophrenia candidate gene (Luo et al., 2019; Sozuguzel et al., 2019). Its relationship with schizophrenia is well supported by linkage or association studies in different populations (van Schijndel et al., 2009; Liu et al., 2010; Wedenoja et al., 2010; Alkelai et al., 2012; Zhou et al., 2016; Tang et al., 2017; Xiao et al., 2017; Sobue et al., 2018). *RELN* is located at 7q22. It encodes the glycoprotein Reelin, which is secreted mainly from the Cajal-Retzius cells and a subpopulation of GABAergic interneurons in the developing cerebral cortex and hippocampus. *RELN* is known as a crucial molecule in brain development, acting as a key regulator in neuronal migration, cell aggregation, dendrite formation, microtubule function, and cell-cell interactions. Animal studies have revealed that *RELN* is an essential molecule for proper cortical neurons migration (Kubo et al., 2010; Franco et al., 2011; Jossin and Cooper, 2011; Kupferman et al., 2014; Sekine et al., 2014; Kohno et al., 2015), and it acts as the final regulator for the cell positioning in the cortex during embryonic and early postnatal stages. However, the expression patterns and distribution of *RELN* in the postnatal period are dramatically changed as compared to those during embryonic period. Intriguingly, evidence has proven that Reelin signaling modulates synaptic function in the adult brain (Alcantara et al., 1998; Herz and Chen, 2006) suggesting *RELN* also plays an important role in postnatal brain. *RELN* is also involved in signaling pathways related to neurotransmission, memory formation, and synaptic plasticity. Studies have shown that Reelin signaling plays a role in the processes of dendrite development (Olson et al., 2006; Jossin and Goffinet, 2007). *RELN* is essential for proper functional and behavioral development of juvenile prefrontal circuits through modulating the N-methyl-D-aspartate receptor (NMDAR) mediated signaling pathway (Iafrati et al., 2014; Lane-Donovan et al., 2015). Furthermore, Reelin signaling is also involved in the presynaptic functions. RELN acts presynaptically in mature neurons to rapidly enhance neurotransmitter release. It has been reported that the Reelin pathway controls learning and memory through activation of the transcriptional factors (Telese et al., 2015). Moreover, it has been shown that variants of *RELN* are closed associated with increased risk of schizophrenia (Shifman et al., 2008). Evidence has implicated the etiology of schizophrenia with regard to the crucial role of *RELN* in neurodevelopment (Fatemi, 2005). Both the mRNA and protein levels of *RELN* have been shown to be markedly reduced in schizophrenia patients (Impagnatiello et al., 1998).

Population studies have indicated that *RELN* may contribute to the genetic etiology of schizophrenia, and the known functions of *RELN* have implicated its involvement in etiology of schizophrenia with regard to a disruption in neurodevelopmental processes (Fatemi, 2005). As mentioned above, most pharmacogenetics studies mainly focus on drug targets genes, drug transporters genes, or cytochrome P450 genes. Therefore, in this study we aim to determine whether *RELN* affects antipsychotic drug treatment outcomes in the Chinese population. This study has the potential to identify novel antipsychotic drug response markers. Moreover, since *RELN* is crucial in the genetic etiology of schizophrenia, our study could find new possibilities for novel drug targets for schizophrenia.

## Materials and Methods

### Subjects

Male and nonpregnant, nonlactating female subjects 18 to 65 years of age with a DSM-IV diagnosis of schizophrenia inpatients, were eligible for the study. All the patients were recruited from Shanghai Mental Health Center. In total, 260 subjects were recruited in this study with a Diagnostic and Statistical Manual of Mental Disorders-IV criteria for schizophrenia. The enrolled subjects were prescribed single antipsychotic medication during the study, including risperidone (150), quetiapine (37), aripiprazole (34), olanzapine (10), and perphenazine (29). The enrolled patients were required to undergo drug cleaning if they were taking antipsychotics medication or withdrawal time is less than the required drug cleaning period. The inclusion criteria were drug naïve or drug cleaning period longer than five metabolic half-life periods and physically healthy with all laboratory parameters within normal limits. Major exclusion criteria were: physical complication or other substance abuse; history suggesting resistance to antipsychotic treatment; significant risk of suicidal or violent behavior, clinically significant abnormal vital signs or laboratory values; uncontrolled major medical illnesses, ischemic heart disease, history of myocardial infarction, coronary bypass surgery, and coronary angioplasty. The study protocol was reviewed and proved by the Shanghai Ethical Committee of Human Genetic Resources. Statement of informed consent was obtained from all subjects after full explanation of the procedure.

### Variant Selection

We selected potential polymorphisms which may be involved in antipsychotic drug responses and were previously associated with schizophrenia. The SNPs were selected based on the following criteria: (1) SNPs previously reported associated with disease pathogenesis; (2) minor allele frequency of >0.1. In total, 15 variants were selected based on systematical literature and database search (dbSNP, HapMap, 1000 Genome). The genomic information of these 15 selected SNPs is listed in **Table 1**.

**Table 1:** Genomic information of selected SNPs of *RELN* genotyped in this study.

SNPs	Allele		Frequency^a^(1000 genome)	Position^b^	Information
	Major	Minor			
rs362719	C	A	0.417	chr7:103545430	Intron variant
rs11496125	C	T	0.405	chr7:103777110	Intron variant
rs155333	T	A/C	0.250	chr7:103798667	Intron variant
rs2237628	T	C	0.478	chr7:103581859	Intron variant
rs2535764	C	G/T	0.270	chr7:103552085	Intron variant
rs362626	A	C/T	0.264	chr7:103576772	Intron variant
rs362726	T	C	0.474	chr7:103566787	Intron variant
rs362731	T	C	0.474	chr7:103568581	Intron variant
rs362814	T	A	0.262	chr7:103574673	Intron variant
rs3808035	A	C/T	0.466	chr7:103513015	Intron variant
rs3819479	T	A/C	0.251	chr7:103756635	Intron variant
rs6465938	T	C	0.415	chr7:103851896	Intron variant
rs7341475	G	A	0.149	chr7:103764368	Intron variant
rs12705169	T	C/G	0.178	chr7:103936441	Intron variant
rs362813	T	A/C	0.474	chr7:103574493	Intron variant
rs39339	T	G	0.118	chr7:103819488	Intron variant

^a^On the basis of 1000 genome. ^b^On the basis of GRCh38.p12.

### Clinical Assessment

For the risperidone, quetiapine, aripiprazole, olanzapine, and perphenazine subjects, the initial dosages were 1, 100–200, 10, 5, and 4 mg/day, respectively. The dosages were gradually increased to 2–6, 400–800, 10–30, 5–20, and 20–60 mg/day within the first week, respectively. The dosages were maintained until the end of week 2. After that, the dosages were adjusted according to individual tolerance. For all the participants, medication compliance was closely monitored and confirmed by the nursing staff, and no other medication was given except trihexylphenidy for extrapyramidal side effects, clonazepam or lorazepam for insomnia, and sennoside for constipation during the 6-week study period.

Clinical effect was assessed on the Positive and Negative Syndrome Scale (PANSS), including the positive, negative and general psychopathology subscales. For all the recruited patients, clinical assessments were conducted on the day of admission, as well as in the treatment process. All PANSS ratings were conducted independently by two qualified psychiatrists who were blind to the genotype of patients. The inter-rater reliability between the two psychiatrists was good. Measurements of psychiatric efficacy, safety, and tolerability were performed at screening, baseline, and the end of weeks 1, 2, 4, and 6, and their PANSS scores were recorded. In this study, 6-week PANSS score was used as the PANSS endpoint score as a measure of response.

### DNA Extraction and Genotyping

Genomic DNA was extracted from leukocytes in venous blood using a QiaAmp^®^ Isolation system (Qiagen, Basel, Switzerland) according to manufacturer’s instructions. SNP genotyping was performed using the MassARRAY^®^ SNP IPLEX platform (Agena Bioscience™, San Diego, CA, USA). Detailed information about the primers design and PCR (polymerase chain reaction) conditions is available upon request. Quality control was performed by excluding individual SNPs or samples with genotype call rates less than 95% and SNP assays with poor-quality spectra or cluster plots. Ten percent of samples were randomly tested on the same platform and no inconsistency was found, which ensured the reliability of further data analyses.

### Statistical Analyses

As classifications based on PANSS could reduce sensitivity and the power of statistical tests, we used percentage change on PANSS to assess treatment responses to antipsychotic medications. PANSS is an interval scale ranging from 1 to 7 and does not have a zero point. To avoid incorrect calculations, we subtracted the theoretical minimum (30 for the total score) from the baseline score, resulting in a score range including zero. In this study, 6-week PANSS score was used as the PANSS endpoint score.

$$\begin{array}{l} \text{PANNS percentage change}=\\ (\text{PANNS baseline score-PANNS endpoint score})/\\ (\text{PANNS baseline score}-30)\times 100 \end{array}$$

The statistical power of this study was calculated online (https://clincalc.com/) using evidence-based clinical decision support tools and calculators for medical professionals. The Student’s t-test was carried out using SPSS v20.0 software (IBM, Armonk, NY, USA) to examine clinical variables. In order to control the nongenetic confounding factors, we have investigated the associations of sex, age, baseline PANSS score, drug-exposure status, and duration of the disorder with response to the antipsychotics. We applied univariate general linear model analyses using SPSS v20.0 software to assess the associations. The association of genotype with antipsychotic response was assessed using general linear model with age as covariate using SPSS v20.0 software. Haploview v4.0 was used to conduct the Hardy–Weinberg equilibrium test and the haplotype analyses. All tests were two-tailed and statistical significance was assumed at p ≤ 0.05. Multivariate interactions were analyzed on multifactor dimensionality reduction (MDR) software. During the MDR process, the data were randomly divided into 10 equal parts, and the MDR was developed on 9/10 of the data (training set) and then tested on 1/10 of the remaining data (testing set). Statistical significance of testing-balanced accuracy of each selected multifactor model was determined by comparing the average prediction error from the observed data with the distribution of average prediction errors under the null hypothesis of no association derived empirically from 1000 permutations. The null hypothesis was rejected when the upper-tail Monte Carlo P-value derived from the permutation test was ≤0.05. SNPStats (Institut Català d’Oncologia, 2006) (https://www.snpstats.net) was used to evaluate the risk under five inheritance models, namely, codominant, dominant, recessive, overdominant, and additive models. All the statistical tests were two-sided, and p < 0.05 was defined as statistically significant.

## Results

### Patient-Related Demographic Information and Clinical Parameters

Of the 260 subjects that formed the study cohort, the duration of the disorder was between 0–42 years. The average duration of the disorder for male was 7.73, and for female was 10.13. In order to assess whether duration of the disorder would influence patients’ response to the antipsychotic drugs, we applied univariate general linear model to assess the association with response to the antipsychotic treatment. No significant difference was noted (p = 0.610). Thus, duration of the disorder was not counted as a confounding factor in our study. We also included drug-exposure status as a covariate to investigate the association with response to the antipsychotics. The percentage of patients who were drug naive was 27.78%. No significant difference between association of drug-exposure status with response was noted (p = 0.586) in our study. Descriptive statistics for patient-related variables such as age and PANSS scores with regard to response in antipsychotic drugs are summarized in **Table 2**. No significant differences in age and baseline PANSS score between the two groups were noted. However, there was a significant difference in age between these two groups. In order to control this confounding factor, we applied general linear model setting age as a covariate to assess the association of genotype with antipsychotic treatment response (PANSS percentage change) in the subsequent analysis. The Hardy–Weinberg equilibrium test showed no significant deviation in the cohort.

**Table 2:** Descriptive statistics for patient-related variables with regard to response.

Response	Sex	*p* ^a^	Age	*p* ^b^	Baseline PANSS score	*p* ^b^
Good responders	Male (54%) Female (46%)	0.405	33.60 ± 11.07	**0.001**	84.65 ± 14.39	0.412
Poor responders	Male (56.87%) Female (43.12%)		39.15 ± 14.06		82.82 ± 17.02	

^a^Chi-square test, p-value from Fisher’s. ^b^Two-tail t-test. p-values in bold indicate significant for the association.

A reduction in total PANSS scores ≥50% were classified as good responders, while others were poor responders. Among the 260 subjects, 40% were good responders (100 patients), and the remaining 60% were poor responders (160 patients). The proportion of good responders for risperidone (150), quetiapine (37), aripiprazole (34), olanzapine (10), and perphenazine (29) were 59.33%, 62.16%, 52.94%, 80%, and 75.86%, respectively.

### Statistical Power

Given the parameters from this study, a power calculation indicated that the study was sufficiently powered (96.5%) to detect many of the SNPs with the present sample size. Since it is commonly used for evaluating statistical power of an existing study, post-hoc Power Calculator model was chosen for this study. The incidence of good response in our study was 38.46%. With the study statistical parameters, we had 96.5% power to detect SNPs at a significance level of 0.05.

### Effects of *RELN* Gene Polymorphisms on Antipsychotic Treatment Response

General linear model analyses setting age as a covariate were carried out to investigate the association of percentage change on PANSS scores with different genotypes. Genotype frequencies of the 15 SNPs and the associations with antipsychotic treatment response are listed in **Table 3**. The analysis showed that two SNPs had significant associations with reduction in PANSS scores, specifically, rs155333 at a significant level of 0.010 and rs6465938 at a borderline significance of 0.049. However, neither of the associations remained significant after Bonferroni correction (*p*-value×15>0.05).

**Table 3:** Association of *RELN* gene polymorphisms with risperidone response.

SNP	Genotype			MAF^a^	MS^b^	F	*P*^c^
rs362719	AA	CC	CA	A: 0.33	0.081	0.313	0.732
	22 (0.088)	107 (0.428)	121 (0.484)				
rs7341475	GG	AA	GA	A: 0.10	0.006	0.023	0.978
	185 (0.811)	1 (0.005)	42 (0.184)				
rs155333	CC	TT	TC	T: 0.22	1.173	4.754	**0.010**
	135 (0.595)	10 (0.044)	82 (0.361)				
rs39339	TT	GG	GT	G: 0.07	0.046	0.173	0.841
	220 (0.870)	1 (0.004)	32 (0.126)				
rs6465938	CC	TT	CT	T: 0.39	0.774	3.045	**0.049**
	88 (0.367)	33 (0.138)	119 (0.495)				
rs3819479	TT	AA	AT	A: 0.22	0.168	0.639	0.529
	145 (0.599)	8 (0.033)	89 (0.368)				
rs3808035	AA	CC	CA	A: 0.38	0.091	0.365	0.695
	88 (0.368)	29 (0.121)	122 (0.511)				
rs2535764	CC	TT	CT	T: 0.18	0.444	1.731	0.179
	161 (0.685)	11 (0.047)	63 (0.268)				
rs12705169	TT	GG	GT	G: 0.16	0.137	0.519	0.596
	180 (0.711)	10 (0.040)	63 (0.249)				
rs2237628	TT	CC	CT	C: 0.48	0.464	1.847	0.160
	50 (0.208)	58 (0.242)	132 (0.550)				
rs362626	AA	CC	CA	A: 0.34	0.198	0.934	0.394
	28 (0.120)	103 (0.442)	102 (0.438)				
rs362814	TT	AA	AT	T: 0.34	0.007	0.027	0.973
	29 (0.120)	105 (0.436)	107 (0.444)				
rs362813	CC	TT	CT	T: 0.47	0.022	0.102	0.903
	66 (0.263)	50 (0.199)	135 (0.538)				
rs362731	TT	CC	CT	C: 0.49	0.475	2.028	0.134
	52 (0.226)	58 (0.252)	120 (0.522)				
rs362726	TT	CC	TC	C: 0.44	0.038	0.149	0.862
	77 (0.231)	49 (0.204)	114 (0.475)				

^a^MAF, minor allele frequency. ^b^MS, mean square. ^c^Analysis control for age. p-values in bold indicate significant for the association.

Case-control study based on the reduction in the PANSS score was also performed to investigate the association of single polymorphism with antipsychotic drug response. At the end of 6-week treatment, 100 patients showed a reduction of ≥50% in total PANSS scores and they were classified as good responders. The remaining 160 patients were classified as poor responders. However, no significant association of antipsychotic drug response was identified using case-control study.

### Association of Haplotypes With Antipsychotic Treatment Response

**Figure 1:**
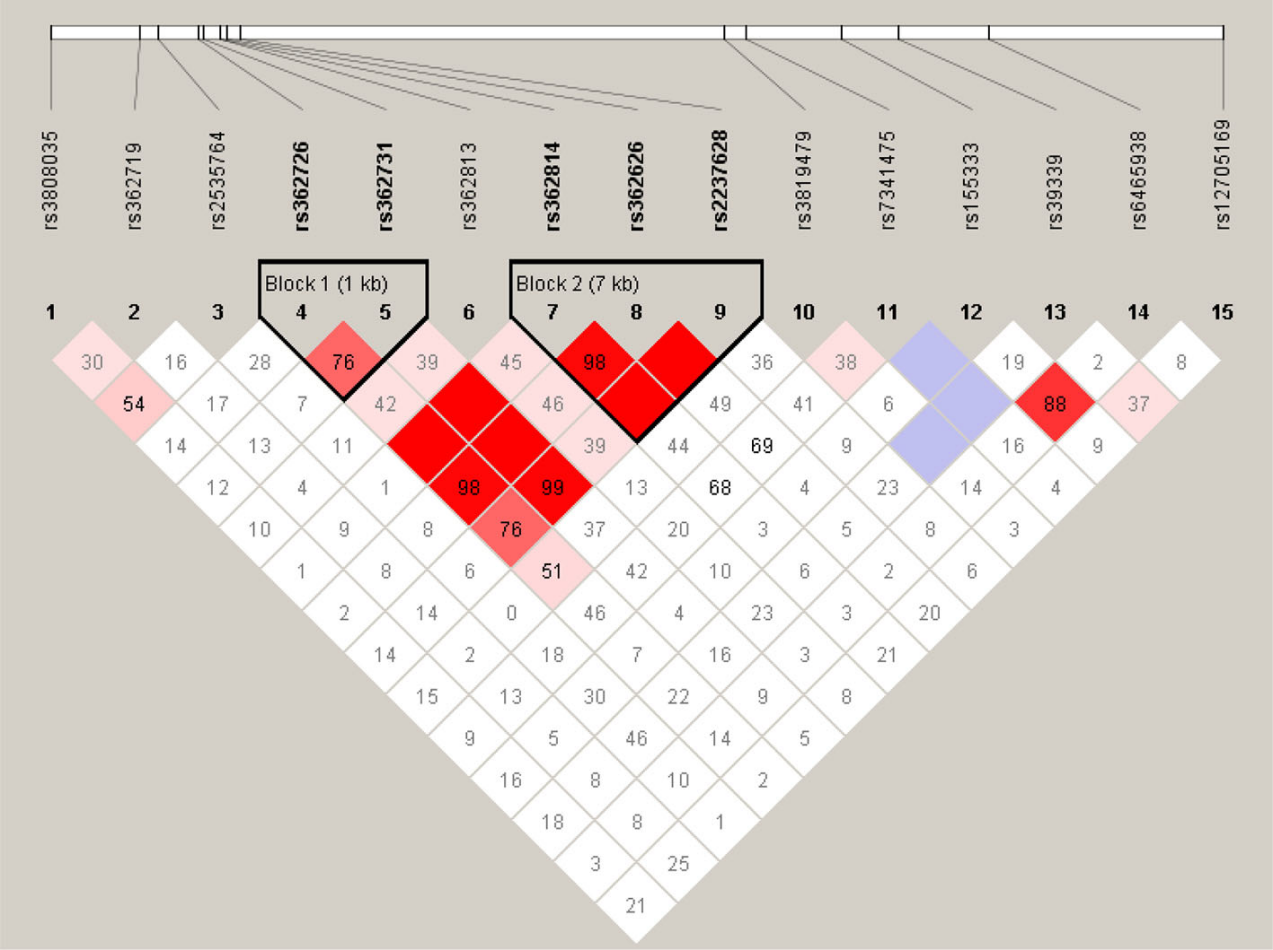
Linkage disequilibrium block structure across RELN gene. The figures show the output of Haploview (version 4.0) LD Plot where each square (with D´ values written within the box) represents a pair-wise LD relationship between the two SNPs. Red squares indicate statistically significant LD between the pair of SNPs as measured by the D’ statistic. Darker colors of red indicate higher values of D’, up to a maximum of 1. White squares indicate pair wise D’ values less than one with no statistically significant evidence of LD.

We performed linkage disequilibrium analysis between each pair of all the 15 SNPs of *RELN* in our cohort and defined the blocks to evaluate haplotype association with the criteria of minor allele frequency >5% and D’ >0.75. Two blocks were identified under the definitions (**Figure 1**). High LD was observed between SNPs rs362726-rs362731 and there was a block consisting of three SNPs (rs362814, rs362626, and rs2237628) ranging 7KB in the chromosome. The subjects were classified into two groups according to reductions in the PANSS scores: good responders and poor responders. Furthermore, comparison of overall frequency differences across all possible haplotypes between good and poor responder groups were carried out (**Table 4**). To further assess haplotype association with treatment response, association analysis of the two haplotypes between good and poor responders was also performed. The results revealed significant association of a haplotype consisting of three SNPs rs362814-rs362626-rs2237628 with antipsychotic treatment response (**Table 4**). Even after 1000 times permutations, the *p*-value indicated significant association (rs362814-rs362626-rs2237628:ACT, χ^2^ = 6.353, *p* = 0.0117, permuted *p* = 0.04. Haplotype ACT (rs362814-rs362626-rs2237628) was more prevalent in poor responders than in good responders.

**Table 4:** Frequency distribution and association analysis of haplotypes of *RELN* gene with antipsychotics response.

Haplotype			Haplotype frequency	Good responder (%)	Poor responder (%)	χ^2^	*p*-value^a^	*p*-value^b^
rs362726	rs362731							
T	T		0.440	0.411	0.457	1.003	0.3167	0.913
C	C		0.394	0.415	0.382	0.541	0.462	0.978
T	C		0.115	0.128	0.108	0.461	0.4973	0.984
C	T		0.050	0.045	0.053	0.132	0.7164	0.999
rs362814	rs362626	rs2237628						
A	C	C	0.519	0.548	0.501	1	0.3173	0.914
T	A	T	0.339	0.357	0.328	0.44	0.5072	0.985
A	C	T	0.134	0.084	0.164	6.353	**0.0117**	**0.04**

^a^Uncorrected p-value. ^b^Permuted p-value, number of permutations: 1000. p-values in bold indicate significant for the association.

### Multivariate Interaction Analysis of Antipsychotic Drug Response

Multifactor dimensionality reduction (MDR) analyses were used to investigate probable multivariate interactions (including all SNPs, sex, age, weight, *etc.*) associated with antipsychotic drug treatment response. MDR is a model-free and non-parametrical approach method that can identify high dimensional gene-gene or gene-environment interactions in populations. Two-locus interactions through four-locus interactions were analyzed in the present study. Training-balanced accuracy and testing-balanced accuracy were obtained for each selected model. The multifactor model with the best testing-balanced accuracy and cross-validation consistency was selected. The best models are summarized in **Table 5**. However, the permutation testing showed that the two-, three- and four-locus best model was not significant associated with antipsychotic drug response, suggesting that there were no interactions between the 15 SNPS.

**Table 5:** Results of the MDR analysis of the dataset.

SNPs included in the best candidate model	Training Bal. Acc.	Testing Bal. Acc.	Cross- validation consistency	*p*-value^a^
rs3819479, rs362626	0.6146	0.4594	3/10	0.9880
rs362719, rs3808035, rs362726	0.6806	0.4775	5/10	0.9570
sex,rs362719, rs3808035, rs362726	0.7453	0.3938	3/10	0.9990

Acc, accuracy; Bal, balanced; MDR, multifactor dimensionality reduction. ^a^p-value based on 1000 permutations.

### Five Genetic Models Analysis

We further investigated the 15 SNPs relations to antipsychotic drug response in five genetic models while controlling for confounding factors. The subjects were classified into two groups according to reductions in the PANSS scores: good responders and poor responders. The adjustment for age and gender factors in the case-control samples was executed using unconditional logistic regression under five inheritance models. Notably, rs2535764 was found to be associated with antipsychotic drug response under overdominant genetic model at a marginal significant level of 0.046 (C/T vs. C/C + T/T: *p* = 0.046, AIC = 314.7, BIC = 321.6). Genotype C/T were more prevalent in good responders than in poor responders. Genotype C/C and T/T was associated with a poor response risk in the study population (OR = 0.55, P value = 0.046). Even after adjusting for age and sex, the associations remained significant in this model (**Table 6**).

**Table 6:** Logistic regression analysis of associations between the genotypes of *RELN* rs2535764 with antipsychotic response.

Model	Genotype	Good responder (%)	Poor responder (%)	OR (95% CI)	P-value	AIC	BIC
Codominant	C/C	56 (62.9%)	106 (71.1%)	1.00	0.069	315.3	325.7
	C/T	31 (34.8%)	34 (22.8%)	0.58 (0.32–1.04)			
	T/T	2 (2.2%)	9 (6%)	2.38 (0.50–11.38)			
Dominant	C/C	56 (62.9%)	106 (71.1%)	1.00	0.19	316.9	323.9
	C/T-T/T	33 (37.1%)	43 (28.9%)	0.69 (0.39–1.20)			
Recessive	C/C-C/T	87 (97.8%)	140 (94%)	1.00	0.16	316.6	323.6
	T/T	2 (2.2%)	9 (6%)	2.80 (0.59–13.25)			
Overdominant	C/C-T/T	58 (65.2%)	115 (77.2%)	1.00	**0.046**	314.7	321.6
	C/T	31 (34.8%)	34 (22.8%)	0.55 (0.31–0.99)			
Log-additive	—	—	—	0.87 (0.55–1.38)	0.56	318.3	325.3

AIC, Akaike Information Criterion. BIC, Bayesian information criterion. p-values in bold indicate significant for the association.

## Discussion

*RELN* plays an important role in the etiology of schizophrenia. Few studies have investigated whether variants of *RELN* affects antipsychotic drug treatment outcomes. In the present study, we focused on this well-known candidate gene to evaluate the role of *RELN* in response to antipsychotic treatment response. Fifteen SNPs were selected to investigate their potential as genetic markers to predict antipsychotic treatment efficacy. The major findings were that two novel SNPs of *RELN* (rs155333 [p = 0.010] and rs6465938 [p = 0.049]) were identified to be associated with antipsychotic treatment response. Our study revealed highly significant association of a haplotype consisting of three SNPs rs362814-rs362626-rs2237628 with antipsychotic treatment response. A novel SNP, rs2535764, was also found to be associated with antipsychotic treatment response under the overdominant genetic model. Our data indicates that *RELN* can affect the outcomes of antipsychotics therapies in the Chinese Han population. Our findings suggest that these SNPs have the potential to be used as antipsychotic treatment markers. Combined with previous studies, our findings may provide useful information for future designs of clinically useful predictive biomarkers of antipsychotic drug response.

Our results support our hypothesis that the risk gene of schizophrenia, *RELN,* can affect antipsychotic treatment outcomes in the Chinese population, suggesting that the susceptibility genes might be potential therapeutic targets. These findings have certain clinical significance. Firstly, our findings may provide novel drug response markers to be used by medical stuff to identify if the patients will have generally satisfactory or unsatisfactory treatment responses. Secondly, our findings may lead to the further study of the mechanism of antipsychotics response affected by *RELN* variants. This may lay the foundation for novel drug response markers discovery.

In the present study, we have identified two novel SNPs rs155333 and rs6465938 that were associated with antipsychotic response. One novel SNP rs2535764 was also found to be associated with antipsychotics response under the overdominant genetic model. To the best of our knowledge, this is the first study to report the associations of three SNPs associated with antipsychotic response. Rs155333 is an intronic locus within *RELN*. Han et al. have reported that rs155333 was significantly associated with cognitive impairment at a level of conventional genome-wide significance (*P*_adjusted_  =  1.3 × 10−^8^) (Han et al., 2017). Kahler et al. performed an association study on 839 schizophrenia cases and 1,473 controls of Scandinavian origin. Their study showed that rs155333 of *RELN* attained nominal significant *p*-values (*p* < 0.05) in both genotypic and allelic association test (Kahler et al., 2008). Rs6465938 was also reported to be significantly associated with the risk of schizophrenia in the Scandinavian origin population. The combined rs262355, rs155333, and rs6465938 haplotype was also significantly associated with schizophrenia in that study (*p* = 0.031) (Kahler et al., 2008). These previous findings indicated that rs155333 and rs6465938 genetically contribute to the risk of cognitive function and involve in schizophrenia pathology. Furthermore, our study suggests that rs155333 and rs6465938 could be novel drug targets for schizophrenia.

Previous researches have suggested haplotype-based association methods are more powerful than single locus-based methods (Shifman et al., 2002), therefore, in the present study, haplotype-based association analyses were performed to investigate the effects on antipsychotic treatment response. A single SNP is not sufficient to predict drug response (Drysdale et al., 2000; Luo et al., 2019), however, the interaction of several SNPs in a haplotype can affect the physiological reaction and response to treatment. In this study, the association of *RELN* with antipsychotics response was further supported by the results of haplotype analysis. A haplotype consisting of rs362814-rs362626-rs2237628 in *RELN* showed highly significant association with antipsychotic treatment response (**Table 4**). Even after permutation, the *p*-value indicated significant association (*p* = 0.0117, permuted *p* = 0.04. Haplotype A-C-T was more prevalent in poor responders than in good responders, suggesting that patients with haplotype A-C-T have a high risk of suffering poor antipsychotic treatment response. Several studies have also reported the associations of haplotypes with the risk of schizophrenia (Kahler et al., 2008; Li et al., 2011; Li et al., 2013; Luo et al., 2019). A recent study reported a haplotype consisting of rs362814, rs39339, rs540058, and rs661575 was found to be significantly associated with schizophrenia even after Bonferroni correction (chi(2) = 29.024, *p* = 6.42E-04, *p* Bonf = 0.017), and the T-C-T-C haplotype was a protective factor for schizophrenia (OR = 0.050, 95% CI = 0.004–0.705) (Luo et al., 2019). Li et al. have identified that the haplotypes incorporating the SNPs (rs2237628, rs362626, rs362814, rs362813, rs362731, and rs362726) (Li et al., 2013) were significantly associated with schizophrenia. Our study is the first one to report that a haplotype of rs362814-rs362626-rs2237628 in *RELN* showed highly significant association with antipsychotic treatment response. In clinical practice, the effect size of one SNP as a response-related factor might be too small to predict treatment response. Therefore, the significant haplotype in our study may serve as a better marker to guide clinical individual medication in the future than a single SNP marker.

We also conducted a combined analysis between all the selected SNPs using MDR analysis in the present study. However, no model showed significant association with antipsychotic treatment response, suggesting there was no interaction among these SNPs.

In order to fully mine the data and provide systematic and comprehensive analysis, we applied different strategies for data analysis. They have different algorithms and different merit. In total, we applied five different analysis methods, three for single SNP association analysis (general linear model analyses, chi-square tests, and five genetic models analysis), one for haplotype-based association analysis (linkage disequilibrium analysis), and one for multivariate interactions analysis (MDR model). Using general linear model analyses, we can control the non-genetic confounding factors as covariates to investigate the association of percentage change on PANSS scores with different genotypes. This method is more rigorous. Chi-square tests is another different method for single SNP association analysis. It’s case-control study strategy. The subjects were classified as two groups. Five genetic models analysis is also a method for single SNP association analysis, but the merit is that the data can be analyzed using unconditional logistic regression under five inheritance models (codominant, dominant, recessive, overdominant, and log-additive). Because haplotype-based association methods are more powerful than single locus-based methods, so we also applied linkage disequilibrium analysis to investigate the haplotype consisting of several SNPs associated with drug response. Multifactor dimensionality reduction (MDR) analysis is a more powerful model to investigate probable multivariate interactions, including all SNPs and the non-genetic clinical factors. It can identify high dimensional gene–gene or gene–environment interactions in population.

This study reported the association of *RELN* with antipsychotic response. Our findings may provide novel drug response markers. The potential of using *RELN* as a novel drug target has been investigated. Several studies have directly administrated RELN protein into the mouse brain to evaluate this possibility and examine the effects. Ishii et al. demonstrated that RELN had a preventive effect on phencyclidine-induced behavioral deficits (Ishii et al., 2015). Another group demonstrated that *in vivo* injection of RELN into the mouse cerebral ventricle affected the synaptic and cognitive functions in wild-type mice and heterozygous reeler mice (Rogers et al., 2011; Rogers et al., 2013). This group further showed that RELN administration ameliorated both the synaptic plasticity and the cognitive behavioral deficits in a mouse model of Angelman syndrome, which is characterized by mental retardation, absence of speech, seizures, and motor dysfunction (Hethorn et al., 2015).

Our study also had some limitations. Although we have considered underlying non-genetic factors such as sex, age, baseline PANSS score, drug-exposure, and duration of illness as covariates, other potential factors, such as treatment history, dosage of different antipsychotics, smoking, baseline weight, and concomitant medication should also be considered in future clinical information collection. Our makers were only identified in the Chinese Han population. It needs validation in other ethnicities and in larger sample sizes.

## Conclusion

In summary, the aim of this study was to determine whether risk gene of schizophrenia *RELN* affects antipsychotic treatment outcomes in the Chinese Han population. We have identified two novel genetic loci of *RELN* associated with response to antipsychotic treatment in patients with schizophrenia. Future research should extend these findings to larger samples and different populations to confirm their potential use in the development of personalized medicine.

## Data Availability Statement

The datasets generated for this study will not be made publicly available: the original human genetic resources including genotype is not permitted to share, it is illegal.

## Ethics Statement

The studies involving human participants were reviewed and approved by the Shanghai Ethical Committee of Human Genetic Resources. The patients/participants provided their written informed consent to participate in this study.

## Author Contributions

QX carried out the experiments and wrote this manuscript. ML and YL contributed to the data processing. WY, YF, DZ, DW, SQ, AN and HL contributed to the collection of materials. SY helped to revise the manuscript. QX, SY and WY designed and revised the manuscript.

## Funding

This work was supported by the National Key Research and Development Program of China (2016YFC1307005), Natural Science Foundation of Shanghai (17ZR1424600), Foundation of Shanghai Mental Health Center “Sailing Plan” (2018-QH-05), Foundation of Shanghai Jiao Tong University School of Medicine (16XJ21010), National Science and Technology Major Project for IND (investigational new drug) (2018ZX09734-005), Clinical Research Center, Shanghai Jiao Tong University School of Medicine (DLY201620), Strategic Priority Research Program of Chinese Academy of Sciences (XDA12040105), Foundation of Shanghai Mental Health Center (2017-YJ-16), the 863 Program (2012AA02A515, 2012AA021802), the National Nature Science Foundation of China (81773818, 81273596, 30900799, 81671326), National Key Research and Development Program (2017YFC0909303, 2016YFC0905000, 2016YFC0905002, 2016YFC1200200, 2016YFC0906400), The 4th Three-year Action Plan for Public Health of Shanghai (15GWZK0101), and Shanghai Pujiang Program (17PJD020).

## Conflict of Interest

The authors declare that the research was conducted in the absence of any commercial or financial relationships that could be construed as a potential conflict of interest.

## References

[B1] Alcantara S. Ruiz M. D’Arcangelo G. Ezan F. de Lecea L. Curran T. (1998). Regional and cellular patterns of reelin mRNA expression in the forebrain of the developing and adult mouse. J. Neurosci. 18 (19), 7779–7799. 10.1523/JNEUROSCI.18-1907779.1998 9742148 PMC6792998

[B2] Alkelai A. Lupoli S. Greenbaum L. Kohn Y. Kanyas-Sarner K. Ben-Asher E. (2012). DOCK4 and CEACAM21 as novel schizophrenia candidate genes in the Jewish population. Int. J. Neuropsychopharmacol. 15 (4), 459–469. 10.1017/s1461145711000903 21682944

[B3] Arranz M. J. de Leon J. (2007). Pharmacogenetics and pharmacogenomics of schizophrenia: a review of last decade of research. Mol. Psychiatry 12 (8), 707–747. 10.1038/sj.mp.4002009 17549063

[B4] Drysdale C. M. McGraw D. W. Stack C. B. Stephens J. C. Judson R. S. Nandabalan K. (2000). Complex promoter and coding region beta(2)-adrenergic receptor haplotypes alter receptor expression and predict *in vivo* responsiveness. Proc. Natl. Acad. Sci. United States America 97 (19), 10483–10488. 10.1073/pnas.97.19.10483 PMC27050 10984540

[B5] Fatemi S. H. (2005). Reelin glycoprotein: structure, biology and roles in health and disease. Mol. Psychiatry 10 (3), 251–257. 10.1038/sj.mp.4001613 15583703

[B6] Fijal B. Kinon B. Kapur S. Stauffer V. Conley R. Jamal H. (2009). Candidate-gene association analysis of response to risperidone in African-American and white patients with schizophrenia. Pharmacogenomics J. 9 (5), 311–318 10.1038/tpj.2009.24. 19451915

[B7] Franco S. J. Martinez-Garay I. Gil-Sanz C. Harkins-Perry S. R. Muller U. (2011). Reelin regulates cadherin function *via* Dab1/Rap1 to control neuronal migration and lamination in the neocortex. Neuron 69 (3), 482–497. 10.1016/j.neuron.2011.01.003 21315259 PMC3056352

[B8] Han L. Jia Z. Cao C. Liu Z. Liu F. Wang L. (2017). Potential contribution of the neurodegenerative disorders risk loci to cognitive performance in an elderly male gout population. Med. (Baltimore) 96 (39), e8195. 10.1097/MD.0000000000008195 PMC5626325 28953682

[B9] Herz J. Chen Y. (2006). Reelin, lipoprotein receptors and synaptic plasticity. Nat. Rev. Neurosci. 7 (11), 850–859. 10.1038/nrn2009 17053810

[B10] Hethorn W. R. Ciarlone S. L. Filonova I. Rogers J. T. Aguirre D. Ramirez R. A. (2015). Reelin supplementation recovers synaptic plasticity and cognitive deficits in a mouse model for Angelman syndrome. Eur. J. Neurosci. 41 (10), 1372–1380. 10.1111/ejn.12893 25864922 PMC4676289

[B11] Huang Y. Wang Y. Wang H. Liu Z. Yu X. Yan J. (2019). Prevalence of mental disorders in China: a cross-sectional epidemiological study. Lancet Psychiatry 6 (3), 211–224. 10.1016/S2215-0366(18)30511-X 30792114

[B12] Iafrati J. Orejarena M. J. Lassalle O. Bouamrane L. Chavis P. (2014). Reelin, an extracellular matrix protein linked to early onset psychiatric diseases, drives postnatal development of the prefrontal cortex *via* GluN2B-NMDARs and the mTOR pathway. Mol. Psychiatry 19 (4), 417–426. 10.1038/mp.2013.66 23752244 PMC3965840

[B13] Impagnatiello F. Guidotti A. R. Pesold C. Dwivedi Y. Caruncho H. Pisu M. G. (1998). A decrease of reelin expression as a putative vulnerability factor in schizophrenia. Proc. Natl. Acad. Sci. United States America 95 (26), 15718–15723. 10.1073/pnas.95.26.15718 PMC28110 9861036

[B14] Ishii K. Nagai T. Hirota Y. Noda M. Nabeshima T. Yamada K. (2015). Reelin has a preventive effect on phencyclidine-induced cognitive and sensory-motor gating deficits. Neurosci. Res. 96, 30–36. 10.1016/j.neures.2014.12.013 25573715

[B15] Jann M. W. Penzak S. R. (2018). Long-acting injectable second-generation antipsychotics: an update and comparison between agents. CNS Drugs 32 (3), 241–257. 10.1007/s40263-018-0508-6 29569082

[B16] Jossin Y. Cooper J. A. (2011). Reelin, Rap1 and N-cadherin orient the migration of multipolar neurons in the developing neocortex. Nat. Neurosci. 14 (6), 697–703. 10.1038/nn.2816 21516100 PMC3102785

[B17] Jossin Y. Goffinet A. M. (2007). Reelin signals through phosphatidylinositol 3-kinase and Akt to control cortical development and through mTor to regulate dendritic growth. Mol. Cell. Biol. 27 (20), 7113–7124. 10.1128/Mcb.00928-07 17698586 PMC2168915

[B18] Kahler A. K. Djurovic S. Kulle B. Jonsson E. G. Agartz I. Hall H. (2008). Association analysis of schizophrenia on 18 genes involved in neuronal migration: MDGA1 as a new susceptibility gene. Am. J. Med. Genet. B. Neuropsychiatr. Genet. 147B (7), 1089–1100. 10.1002/ajmg.b.30726 18384059

[B19] Kahn R. S. Fleischhacker W. W. Boter H. Davidson M. Vergouwe Y. Keet I. P. (2008). Effectiveness of antipsychotic drugs in first-episode schizophrenia and schizophreniform disorder: an open randomised clinical trial. Lancet 371 (9618), 1085–1097. 10.1016/S0140-6736(08)60486-9 18374841

[B20] Kahn R. S. Sommer I. E. Murray R. M. Meyer-Lindenberg A. Weinberger D. R. Cannon T. D. (2015). Schizophrenia. Nat. Rev. Dis. Primers 1, 15067. 10.1038/nrdp.2015.67 27189524

[B21] Kirchheiner J. Fuhr U. Brockmoller J. (2005). Pharmacogenetics-based therapeutic recommendations–ready for clinical practice? Nat. Rev. Drug Discovery 4 (8), 639–647. 10.1038/nrd1801 16056390

[B22] Kohno T. Honda T. Kubo K. Nakano Y. Tsuchiya A. Murakami T. (2015). Importance of Reelin C-terminal region in the development and maintenance of the postnatal cerebral cortex and its regulation by specific proteolysis. J. Neurosci. 35 (11), 4776–4787. 10.1523/JNEUROSCI.4119-14.2015 25788693 PMC6605132

[B23] Kubo K. Honda T. Tomita K. Sekine K. Ishii K. Uto A. (2010). Ectopic Reelin induces neuronal aggregation with a normal birthdate-dependent “inside-out” alignment in the developing neocortex. J. Neurosci. 30 (33), 10953–10966. 10.1523/JNEUROSCI.0486-10.2010 20720102 PMC6633482

[B24] Kupferman J. V. Basu J. Russo M. J. Guevarra J. Cheung S. K. Siegelbaum S. A. (2014). Reelin signaling specifies the molecular identity of the pyramidal neuron distal dendritic compartment. Cell 158 (6), 1335–1347. 10.1016/j.cell.2014.07.035 25201528 PMC4183142

[B25] Lane-Donovan C. Philips G. T. Wasser C. R. Durakoglugil M. S. Masiulis I. Upadhaya A. (2015). Reelin protects against amyloid beta toxicity *in vivo* . Sci. Signaling 8 (384). 10.1126/scisignal.aaa6674 PMC4652952 26152694

[B26] Lee S. T. Ryu S. Kim S. R. Kim M. J. Kim S. Kim J. W. (2012). Association study of 27 annotated genes for clozapine pharmacogenetics: validation of preexisting studies and identification of a new candidate gene, ABCB1, for treatment response. J. Clin. Psychopharmacol. 32 (4), 441–448. 10.1097/JCP.0b013e31825ac35c 22722500

[B27] Li W. Song X. Zhang H. Yang Y. Jiang C. Xiao B. (2011). Association study of RELN polymorphisms with schizophrenia in Han Chinese population. Prog. Neuropsychopharmacol. Biol. Psychiatry 35 (6), 1505–1511. 10.1016/j.pnpbp.2011.04.007 21549172

[B28] Li M. Luo X. J. Xiao X. Shi L. Liu X. Y. Yin L. D. (2013). Analysis of common genetic variants identifies RELN as a risk gene for schizophrenia in Chinese population. World J. Biol. Psychiatry 14 (2), 91–99. 10.3109/15622975.2011.587891 21745129

[B29] Liu Y. Chen P. L. McGrath J. Wolyniec P. Fallin D. Nestadt G. (2010). Replication of an association of a common variant in the Reelin gene (RELN) with schizophrenia in Ashkenazi Jewish women. Psychiatr. Genet. 20 (4), 184–186. 10.1097/YPG.0b013e32833a220b 20431428 PMC2901865

[B30] Luo X. Chen S. Xue L. Chen J. H. Shi Y. W. Zhao H. (2019). SNP variation of RELN gene and schizophrenia in a chinese population: a hospital-based case-control study. Front. Genet. 10, 175. 10.3389/fgene.2019.00175 30891068 PMC6413413

[B31] Olson E. C. Kim S. Walsh C. A. (2006). Impaired neuronal positioning and dendritogenesis in the neocortex after cell-autonomous Dab1 suppression. J. Neurosci. 26 (6), 1767–1775. 10.1523/JNEUROSCI.3000-05.2006 16467525 PMC6793623

[B32] Robinson D. G. Gallego J. A. John M. Petrides G. Hassoun Y. Zhang J. P. (2015). A randomized comparison of aripiprazole and risperidone for the acute treatment of first-episode schizophrenia and related disorders: 3-month outcomes. Schizophr. Bull. 41 (6), 1227–1236. 10.1093/schbul/sbv125 26338693 PMC4601722

[B33] Rogers J. T. Rusiana I. Trotter J. Zhao L. Donaldson E. Pak D. T. S. (2011). Reelin supplementation enhances cognitive ability, synaptic plasticity, and dendritic spine density. Learn. Memory 18 (9), 558–564. 10.1101/lm.2153511 PMC3166788 21852430

[B34] Rogers J. T. Zhao L. S. Trotter J. H. Rusiana I. Peters M. M. Li Q. Y. (2013). Reelin supplementation recovers sensorimotor gating, synaptic plasticity and associative learning deficits in the heterozygous reeler mouse. J. Psychopharmacol. 27 (4), 386–395. 10.1177/0269881112463468 23104248 PMC3820099

[B35] Sekine K. Kubo K. Nakajima K. (2014). How does Reelin control neuronal migration and layer formation in the developing mammalian neocortex? Neurosci. Res. 86, 50–58. 10.1016/j.neures.2014.06.004 24969097

[B36] Shifman S. Bronstein M. Sternfeld M. Pisante-Shalom A. Lev-Lehman E. Weizman A. (2002). A highly significant association between a COMT haplotype and schizophrenia. Am. J. Hum. Genet. 71 (6), 1296–1302. 10.1086/344514 12402217 PMC378567

[B37] Shifman S. Johannesson M. Bronstein M. Chen S. X. Collier D. A. Craddock N. J. (2008). Genome-wide association identifies a common variant in the reelin gene that increases the risk of schizophrenia only in women. PloS Genet. 4 (2). 10.1371/journal.pgen.0040028 PMC2242812 18282107

[B38] Sobue A. Kushima I. Nagai T. Shan W. Kohno T. Aleksic B. (2018). Genetic and animal model analyses reveal the pathogenic role of a novel deletion of RELN in schizophrenia. Sci. Rep. 8 (1), 13046. 10.1038/s41598-018-31390-w 30158644 PMC6115412

[B39] Sozuguzel M. D. Sazci A. Yildiz M. (2019). Female gender specific association of the Reelin (RELN) gene rs7341475 variant with schizophrenia. Mol. Biol. Rep. 46 (3), 3411–3416. 10.1007/s11033-019-04803-w 30980267

[B40] Tang J. Fan Y. Li H. Xiang Q. Zhang D. F. Li Z. (2017). Whole-genome sequencing of monozygotic twins discordant for schizophrenia indicates multiple genetic risk factors for schizophrenia. J. Genet. Genomics 44 (6), 295–306. 10.1016/j.jgg.2017.05.005 28645778

[B41] Telese F. Ma Q. Perez P. M. Notani D. Oh S. Li W. B. (2015). LRP8-Reelin-Regulated Neuronal Enhancer signature underlying learning and memory formation. Neuron 86 (3), 696–710. 10.1016/j.neuron.2015.03.033 25892301 PMC4486257

[B42] van Os J. Kapur S. (2009). Schizophrenia. Lancet 374 (9690), 635–645. 10.1016/S0140-6736(09)60995-8 19700006

[B43] van Schijndel J. E. van Loo K. M. J. van Zweeden M. Djurovic S. Andreassen O. A. Hansen T. (2009). Three-cohort targeted gene screening reveals a non-synonymous TRKA polymorphism associated with schizophrenia. J. Psychiatr. Res. 43 (15), 1195–1199. 10.1016/j.jpsychires.2009.04.006 19435634

[B44] Wedenoja J. Tuulio-Henriksson A. Suvisaari J. Loukola A. Paunio T. Partonen T. (2010). Replication of association between working memory and reelin, a potential modifier gene in schizophrenia. Biol. Psychiatry 67 (10), 983–991. 10.1016/j.biopsych.2009.09.026 19922905 PMC3083525

[B45] Xiao X. Yu H. Li J. Wang L. Li L. Chang H. (2017). Further evidence for the association between LRP8 and schizophrenia. Schizophr. Res. 10.1016/j.schres.2017.05.002 28495490

[B46] Xing Q. Gao R. Li H. Feng G. Xu M. Duan S. (2006). Polymorphisms of the ABCB1 gene are associated with the therapeutic response to risperidone in Chinese schizophrenia patients. Pharmacogenomics 7 (7), 987–993. 10.2217/14622416.7.7.987 17054409

[B47] Xu Q. Wu X. Xiong Y. Xing Q. He L. Qin S. (2013). Pharmacogenomics can improve antipsychotic treatment in schizophrenia. Front. Med. 7 (2), 180–190. 10.1007/s11684-013-0249-3 23606027

[B48] Zhou Z. Hu Z. Zhang L. Hu Z. Liu H. Liu Z. (2016). Identification of RELN variation p.Thr3192Ser in a Chinese family with schizophrenia. Sci. Rep. 6, 24327. 10.1038/srep24327 27071546 PMC4829830

